# Measuring and Modeling of Melt Viscosity for Drug Polymer Mixtures

**DOI:** 10.3390/pharmaceutics16030301

**Published:** 2024-02-21

**Authors:** Vincent Kimmel, Enrico Ercolin, Robin Zimmer, Muhammet Yörük, Judith Winck, Markus Thommes

**Affiliations:** 1 Laboratory of Solids Process Engineering, Department of Biochemical and Chemical Engineering, Technical University Dortmund, Emil-Figge-Str. 68, 44227 Dortmund, Germany; vincent.kimmel@tu-dortmund.de (V.K.); enrico.ercolin@tu-dortmund.de (E.E.); robin.zimmer@tu-dortmund.de (R.Z.); muhammet-alpaslan.yoeruek@tu-dortmund.de (M.Y.); judith.winck@tu-dortmund.de (J.W.); 2 INVITE GmbH, Drug Delivery Innovation Center, Chempark Building W32, Otto-Bayer-Str. 32, 51061 Cologne, Germany

**Keywords:** amorphous solid dispersions, rheology, viscosity, plasticizing, filler, drug, polymers, modeling

## Abstract

Melt viscosity is an essential property in pharmaceutical processes such as mixing, extrusion, fused deposition modeling, and melt coating. Measuring and modeling of the melt viscosity for drug/polymer mixtures is essential for optimization of the manufacturing process. In this work, the melt viscosity of nine formulations containing the drug substances acetaminophen, itraconazole, and griseofulvin, as well as the pharmaceutical polymers Eudragit EPO, Soluplus, and Plasdone S-630, were analyzed with a rotational and oscillatory rheometer. The shear rate, temperature, and drug fraction were varied systematically to investigate their influence on viscosity. The results for the pure polymers showed typical shear-thinning behavior and are fundamental for modeling with the Carreau and Arrhenius approaches. The investigations of the viscosity of the drug/polymer mixtures resulted in a plasticizing or a filler effect, depending on the type of drug and the phase behavior. A drug shift factor was proposed to model the change in viscosity as a function of the drug fraction. On this basis, a universal model to describe the melt viscosity of drug/polymer mixtures was developed, considering shear rate, temperature, and drug fraction.

## 1. Introduction

Melt viscosity, which describes the flow resistance of fluids, is an important property in a variety of industries, particularly polymer processing, food, and pharmaceutics [1]. This is because melt viscosity has a major influence on flow behavior through processing equipment such as extruders [2,3,4], 3D printers [5,6,7], or melt coaters [8,9,10]. The viscosity has to be in a certain range for a process to be possible at all. Furthermore, viscosity has a major influence on the energy input of processes and should be optimized for this reason [11,12]. Important factors influencing the viscosity are the temperature, the shear rate, and the type and fraction of additives [13]. Thus, it is important to determine the viscosity using suitable measuring principles. The measuring method determines the measuring range from the very beginning and should be selected appropriately. The measuring principle of the rotational rheometer requires a low amount of material (typically < 1 g). Here, the sample is positioned between a stationary and rotating surface of a given geometry (e.g., plate, cone, cylinder). It has a small measuring range (commonly 10^−2^–10^2^ s^−1^), because increased shear rates can lead to the gap between the measuring surfaces emptying [14]. The measuring principle of an oscillatory rheometer belongs to the category of dynamic mechanical analysis and applies a low-stress oscillating movement to the sample. This measuring method is particularly suitable for determining the viscoelastic properties of polymers. In contrast to the rotational viscometer, where the dynamic viscosity is determined directly, the oscillatory rheometer determines the complex viscosity [15]. The comparability of both measurement methods is subjected to prior testing with the Cox–Merz rule [16]. Furthermore, the sample preparation has a key function in achieving reproducible measurement results, especially with powdered pharmaceutical polymers [17]. Melt viscosity can also be described beyond the range of experimental data with the help of models [18]. They refer to the influencing properties such as shear rate, temperature, and additives.

Typical models for describing the shear rate are Power-Law, Cross, Carreau, and Bird–Carreau–Yasuda [13]. The power-law model expresses viscosity as a power-law function of shear rate and provides a simple but versatile representation for a wide range of fluids, capturing both shear-thinning and shear-thickening behavior. The Carreau and Cross models are similar and offer a smooth transition from Newtonian to power-law behavior as shear rate increases. This makes them particularly suitable for describing shear-thinning polymers, which exhibit a Newtonian behavior at low shear rates. Hence, they are commonly used in industry [19]. The shear-thinning behavior on a molecular level is based on the fact that polymer chains are randomly entangled and slide past each other with difficulty at low shear rates, resulting in high viscosity. Increasing the shear rate disentangles the polymer chains, meaning that they slide past each other more easily, resulting in a lower viscosity [13,14,15]. As an extension, the Bird–Carreau–Yasuda model describes the curvature between Newtonian and shear-thinning behavior more precisely by using an additional parameter [13]. In addition to these classic models, several modifications, like Sisko and Moldflow, were developed and their predictive capacity was compared, resulting in similar accuracy [20]. More complex mixtures of nanoparticle-filled polymers can be characterized using the common Carreau–Yasuda approach [21]. Thus, it is essential to select a model that accurately reflects the fluid properties with an optimum level of complexity.

The temperature dependence of viscosity is usually described using the Arrhenius or Williams–Landel–Ferry models. The Arrhenius model uses an exponential relationship that captures the increase in viscosity with decreasing temperature in a range that is quite distant from the glass transition temperature. A further development of the Arrhenius approach is the Andrade equation, which contains three constants in the modified version [22]. As mentioned before, careful consideration should be made between the complexity of the model, which can lead to overestimation, and the simplicity of the model, which can lead to inaccuracy. In contrast, the Williams–Landel–Ferry model provides a means to characterize the nonlinear increase in viscosity as the material approaches its glass transition [14]. At the molecular level, a low temperature causes a small free volume between the polymer chains, resulting in slower movement of the macromolecules and a decrease of viscosity. With high temperatures, the free volume is larger and the molecules can move more easily, thereby reducing viscosity [15].

When modeling changes to viscosity due to additives, a distinction must be made between miscible and immiscible substances. Most models, including those by Einstein, Maron and Pierce, and Mooney, work well for describing the viscosity of fluids with immiscible additives; this is because there are only repulsive interactions within the polymer, and the viscosity change depends solely on the volume fraction of the filler [23]. Essentially, these models work with a relative viscosity, which describes the viscosity of the polymer-containing immiscible additives in relation to the pure polymer. It has been shown that the relative viscosity frequently increases linearly for low volume fractions (<30 v%) [24,25]. In recent research, the viscosity of solid filled polymers was investigated in relation to the syringeability and injectability of biopharmaceuticals. A linear increase in the logarithm of viscosity with solid concentration was demonstrated, and was described by the Einstein model [26]. In the case, the additive and the polymer are miscible, and a plasticizing effect is often observed. This means that the viscosity is decreasing with as the miscible additive content increases. A number of recent studies show this plasticizing effect of drugs, which are mixed in pharmaceutical polymers [27,28,29]. The approaches of Arrhenius, Bingham, and Grunberg can be used to model miscible polymer mixtures. The viscosity of the pure substances is rated according to the mass fraction [23,30]. However, there are also approaches for normalizing the viscosity of miscible systems to the viscosity of the pure polymer [31]. This normalization is often applied for the zero shear rate viscosity [32]. Further investigations quantified the drug effect to the polymer using a viscosity ratio [31,33]. Another approach to describe the viscosity of mixtures is the correlation of the viscosity with the glass transition temperature [34], which performed successfully up to a drug load up to 10 wt% [35].

However, there is no sharp distinction between the filler and the plasticizing effect, as often discussed in literature. Most often, low drug concentrations in the polymer lead to lower viscosity (plasticizing), while higher concentrations yield in an increase in viscosity (filler). The distinction can be made based on the specific solubility of a drug substance in a certain polymer [36]. When exceeding the solubility, the dissolved drug fraction lowers the viscosity while the suspended drug fraction increases the viscosity. In this way, the drug possesses a superimposed plasticizing and a filler effect simultaneously. These effects were separated using rheology data in order to determine the solubility [37], which were quite in line with results from hot stage microscopy and differential scanning calorimetry. Similar investigations were performed considering the slope in the viscosity curve as function of drug load, which was able to quantify the drug solubility in the polymer [38]. A model that includes all influencing variables of viscosity is not currently available.

In this study, polymers (Eudragit EPO, Soluplus, and Plasdone S-630) were selected, each representing a different polymer class and thus offering a broad spectrum of excipients available on the market. These substances are often used as model excipients in hot melt extrusion processes by various research groups. Therefore, the data obtained are well comparable to the literature. The drugs (acetaminophen, itraconazole, and griseofulvin) selected here are commonly used as model drugs for melt-based processes and also for the investigation of interactions of drug/polymer mixtures with respect to viscosity [39]. Therefore, background information like phase diagrams are available and subsequently utilized in this study [40]. These interactions are reflected in the miscibility and solubility of the drug/polymer systems and could result in a plasticizing effect by decreasing the viscosity [41,42]. Since these effects are highly dependent on the drug/polymer mixtures used, a large number of drug/polymer combinations should be studied.

The purpose of this study was to investigate the melt viscosity of drug/polymer mixtures and to develop a model to predict viscosity as a function of shear rate, temperature, and drug content. From the data generated by an oscillatory rheometer, a model will be developed that predicts viscosity and helps to find the optimal process parameters for melt-flow based processes.

## 2. Materials and Methods

### 2.1. Materials

The basic butylated methacrylate copolymer (bBMA) Eudragit E PO (EPO) (Evonik Industries, Darmstadt, Germany), Soluplus (SOL) (BASF, Ludwigshafen, Germany), and the copovidone (polyvinylpyrrolidone/vinyl acetate, PVPVA) Plasdone S-630 (Ashland, Columbus, OH, USA) were used as polymeric carriers. The drug substances, acetaminophen (ACE) (Caelo, Caesar & Loretz, Hilden, Germany), itraconazole (ITR) (BASF, Ludwigshafen, Germany), and griseofulvin (GRI) (Hawkins, Roseville, MN, USA) served as model drugs and were added to the polymeric carriers to prepare the formulations. Calcium carbonate (CaCO_3_) with a defined particle size of 5 µm (Escal 500, KSL staubtechnik, Lauingen, Germany) was used as an additive to investigate immiscible CaCO_3_/polymer systems.

### 2.2. Sample Preparation Method for Rheological Measurements

Initially, the physical mixtures of drugs and polymers were blended in a mini ball mill Pulverisette 23 (Fritsch, Idar-Oberstein, Germany). The frequency of the ball mill was 50 Hz and the powder was blended three times for three minutes with a one-minute break to prevent plasticizing due to heat generation. This step was skipped for the pure polymers. After blending, vacuum compression molding (VCM) with a 25 mm cylindrical chamber (MeltPrep, Graz, Austria) was utilized to generate reproducible, homogeneous, and air bubble-free samples for rheological measurements [17]. The temperature for plasticizing pure polymers was set 63 K above the glass transition temperature $T_{g}$ for 12 min. This value was selected, based on advice from the MeltPrep manufacturer [43], to ensure the polymer was sufficiently plasticized but below the degradation temperature. For the physical mixtures containing drugs, the temperature at the VCM was set to 10 K above the melt temperature of the drug with the goal of forming a single-phase system. An exception to this was the GRI formulations because the melting temperature of the drug is close to the degradation temperature of the polymers; thus, a temperature of 190 °C was selected. After heating, the vacuum chamber was placed on a cooling plate with a fast-cooling option until the temperature decreased below the glass transition temperature $T_{g}$, and was then stored until further use in a desiccator.

### 2.3. Rheology Measurement Method

The viscosity measurements for the rotational and oscillatory methods were performed using a Haake Mars 60 rheometer (Thermo Fischer Scientific, Karlsruhe, Germany) utilizing samples prepared by vacuum compression molding (VCM). This was equipped with an electrical temperature control module for the bottom plate (TM-EL-P, Thermo Fischer Scientific, Karlsruhe, Germany) and an active upper temperature control cover (TM-EL-H, Thermo Fischer Scientific, Karlsruhe, Germany) above the upper plate. A plate-plate geometry (P20 CS L, Thermo Fischer Scientific, Karlsruhe, Germany) with a 20 mm diameter and a gap of 1 mm was utilized. Before the measurements, both plates were heated up to the same temperature, which was used in the VCM, and the temperature was equilibrated for 15 min. Afterwards, the axial zero point was calibrated, and a temperature-gap function was used to compensate for thermal expansion at different temperatures. Finally, the sample film was placed on the rheometer and equilibrated for 12 min as part of the sample preparation step. The gap between the plates was adjusted and the excess melt was removed. All rheological measurements were performed with a controlled shear rate for the rotational measurements and controlled angular frequency for the oscillatory measurements, as these are the deriving parameters. The rotational measurements were performed in a shear rate range of 10^−1^ to 100 s^−1^ with logarithmic spacing, and the temperature was varied in the range of 130 to 200 °C. The reciprocal shear rate was held constant between each step until the flow equilibrated. The oscillatory measurement mode is generally divided into two steps. In the amplitude sweep, the angular frequency is kept constant, and the deformation amplitude is varied logarithmically from 0.1 to 100% with regards to the gap height. The aim of this test was to find a deformation amplitude in the linear-viscoelastic region (LVE). The laws of elasticity and viscosity by Hooke and Newton for the viscoelastic behavior of polymer melts can only be applied in this region. Beyond the linear-viscoelastic region, the sample undergoes irreversible structural changes, which is outside the scope of the basic law of rheology. Amplitude sweeps were performed first at the limits of the measurement range to confirm the linear-viscoelastic region. Furthermore, it was assumed that the deformation amplitude at the limits of the measurement range fulfilled the requirements for the linear-viscoelastic range in the entire measurement range. Finally, a deformation amplitude of 1% with regards to the gap height was determined for all further frequency sweep measurements. Within the frequency sweep, the complex viscosity $\left(\left|\eta^{*}\left(\omega \right)\right|\right)$ depending on the angular frequency $\left(\omega \right)$ was determined. The angular frequency was varied, starting at 0.628 to 628 rad s^−1^, and the temperature was varied in a range of 130 to 200 °C. All measurements were performed in triplicate.

## 3. Results and Discussion

### 3.1. Viscosity Curves and Modeling for Pure Polymers

**Figure 1:**
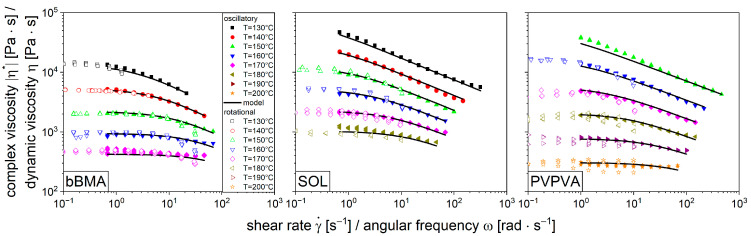
Superposition of viscosity curves from oscillatory (filled symbols: complex viscosity) and rotational (unfilled symbols: dynamic viscosity) measurements from pure polymers EPO (**left**), SOL (**middle**), and PVPVA (**right**) for temperatures 130 °C to 200 °C depending on angular frequency or shear rate. Solid lines show the fitted model of the Carreau–Arrhenius approach (*n* = 3).

Polymer melts are often measured using oscillatory rheometry rather than performing rotational measurements. This usually leads to more reliable determinations of viscoelastic properties [15] but also extends the observable range to higher shear rates [39], which is relevant for many technical applications. In order to apply common rheological laws to the data obtained by oscillatory rheology, a correlation of the dynamic viscosity $\left(\eta \left(\dot{\gamma }\right)\right)$ with the complex viscosity obtained by oscillatory rheometry was performed. Initial investigations dealt with the rheological behavior of the pure polymers (bBMA, SOL, and PVPVA), which served for comparison. Therefore, the viscosity was measured in rotational and oscillatory mode as a function of temperature (Figure 1) and the raw data were enclosed in the Supplementary Materials.

Since the dynamic viscosity (unfilled symbols, Figure 1) and the complex viscosity (filled symbols, Figure 1) measurements were quite comparable for all pure polymer results, the Cox–Merz rule (Equation (1)) was found to be applicable. Thus, all models that apply to the dynamic viscosity can also be applied to the complex viscosity [16].

(1) $$\eta \left(\dot{\gamma }\right)=\left|\eta^{*}\left(\omega \right)\right|$$

With the rotational mode, only small shear rates of up to 10 $s^{-1}$ could be measured as gap emptying occurred. Since higher shear rates are more relevant for technical applications, the oscillatory mode was used for further investigations. For all polymers, the complex viscosity decreased with increasing angular frequency. This shear-thinning behavior is common for polymer melts and related to the molecular alignment upon shear [13]. Furthermore, all viscosity curves were lowered with increasing temperature, which is related to thermal expansion leading to higher molecular mobility by the increase of specific volume [15,23]. The repetitive measurements (*n* = 3) are invisible since the data points are on top of each other (Figure 1).

The shear-thinning behavior of the polymers was described by the Carreau model (Equation (2)) [15]. This model is commonly used because it represents the typical viscosity curve of molten polymers [23]. There are three parameters used to describe the dynamic viscosity $\left(\eta \right)$ as function of shear rate $\left(\dot{\gamma }\right)$: the zero shear rate viscosity $\left(\eta_{0}\right)$, which describes the plateau at low shear rates, the critical shear rate $\left({\dot{\gamma }}_{c}\right)$, which is the shear rate where the shear-thinning behavior begins, and the flow index $\left(c\right)$, which represents the negative slope of the shear-thinning section in double-logarithmic scale.

(2) $$\eta \left(\dot{\gamma }\right)=\frac{\eta_{0}}{{\left(1+\frac{\dot{\gamma }}{{\dot{\gamma }}_{c}}\right)}^{c}}$$

In order to account for different temperatures, the time-temperature superposition approach was utilized, with the required shift factor determined according to Arrhenius (Equation (3)) [14]. The temperature shift factor $\left(a_{T}\left(T,T_{ref}\right)\right)$ is the ratio of zero shear rate viscosities at different temperatures $\left(\eta_{0}(T)/\eta_{0}(T_{ref})\right)$ and correlated to reciprocal temperature on a logarithmic scale. A linear function is obtained where the slope is interpreted as a ratio between the energy of activation $\left(E_{A}\right)$ and the ideal gas constant $\left(R\right).$ The more universal Williams–Landel–Ferry (WLF) approach was not utilized because the temperature for the viscosity measurements was far away from the glass transition temperature. Thus, the shift factor still correlated linearly with respect to the reciprocal temperature, so the Arrhenius approach could be applied [13].

(3) $$a_{T}\left(T,T_{ref}\right)=\frac{\eta_{0}(T)}{\eta_{0}(T_{ref})}=exp\left(\frac{E_{A}}{R}\cdot \left[\frac{1}{T}-\frac{1}{T_{ref}}\right]\right)$$

Coupling the Carreau approach to the time-temperature superposition concept leads to Equation (4) [23], which combines Carreau with Arrhenius to describe the shear rate as well as the temperature dependency of the viscosity.

(4) $$\eta \left(\dot{\gamma },T\right)=\frac{\eta_{0}\cdot a_{T}\left(T,T_{ref}\right)}{{\left(1+a_{T}\left(T,T_{ref}\right)\cdot \frac{\dot{\gamma }}{{\dot{\gamma }}_{c}}\right)}^{c}}$$

**Figure 2:**
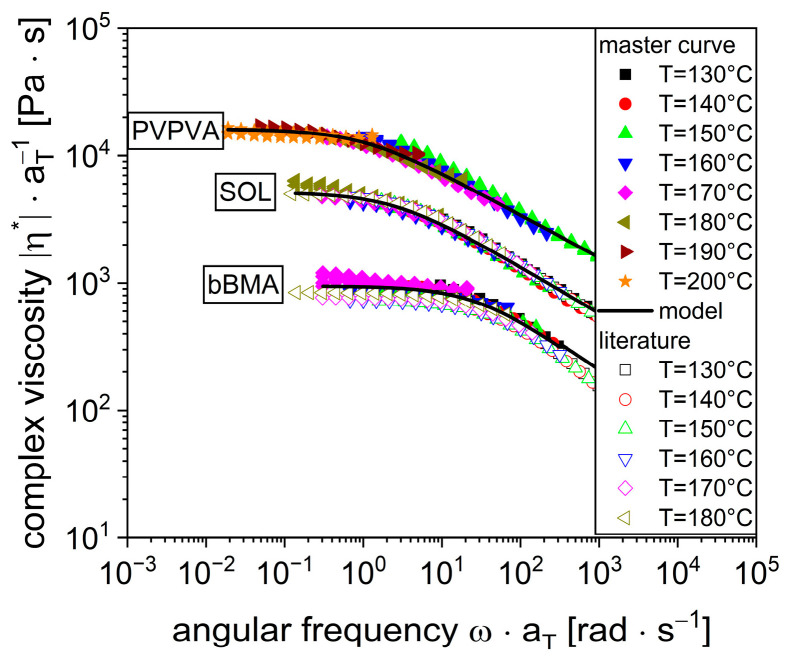
Master viscosity curves of pure polymers bBMA, SOL, PVPVA as measured (filled symbols) and in comparison to literature values [17] for the polymers bBMA and SOL (unfilled symbols) reference temperature T_ref_ = 160 °C.

The concept of shifting the viscosity to a master curve is used to determine the parameters of this equation. Initially, the viscosity curves (Figure 1) are shifted along lines of constant shear stress to a reference temperature of 160 °C until they lie on top of each other with the help of the time-temperature superposition (Equation (3)). This superposition of the individual viscosity curves to one single master curve for each polymer is shown in Figure 2.

Subsequently, the shifted experimental data were used to determine the parameters $\left(\eta_{0},{\dot{\gamma }}_{c},c,E_{A}\right)$ of the combined Carreau–Arrhenius model simultaneously (Equation (4)) using MATLAB (Version R2019b). The MATLAB function fmincon [44] was utilized, which finds the minimum of constrained, non-linear, multivariable functions. In this way, a standard deviation for the individual parameters, based on the repeated measurements, was obtained, resulting in the parameters in Table 1.

**Table 1:** Viscosity parameters of the Carreau–Arrhenius model of pure polymers bBMA, SOL, PVPVA at reference temperature, T_ref_ = 160 °C, as $\bar{x}\pm s$ (*n* = 3), as well as the literature values for bBMA and SOL [17].

Pure Polymer	bBMA	bBMA [17]	SOL	SOL [17]	PVPVA
$\eta_{0}[Pa\cdot s]$	1011 ± 34	772	5146 ± 93	5063	18,296 ± 486
${\dot{\gamma }}_{c}[s^{-1}]$	26.8 ± 5.5	52.0	2.5 ± 0.1	4.5	1.0 ± 0.0
c[−]	0.433 ± 0.018	0.543	0.363 ± 0.002	0.416	0.368 ± 0.003
$E_{A}[kJ\cdot {kg}^{-1}]$	122,498 ± 801	128,761	130,846 ± 344	139,821	184,119 ± 2442

Even though there are many rheological measurements published for these polymers, the results are not readily comparable due to different sample preparation methods, various measuring procedures, and several modeling approaches. There is one study where vacuum compression molding is also used for comparison [17]. Therefore, the viscosity data from the flow function were reevaluated using the aforementioned procedure in order to enable a comparison. The literature data represented by the open symbols (Figure 2) are quite comparable to the results of the current investigation for bBMA and SOL. Slightly lower viscosity values are given in the literature, which could be attributed to batch variability of the polymer or difference in the adsorbed water vapor. However, the experimental difference between the two studies is similar to the experimental error within this study, considering different temperatures. Additionally, the parameters of the Carreau–Arrhenius model for the literature data were determined (Table 1) to be quite similar even if slight deviations can be seen. These models are shown as solid black lines in Figure 1 for the viscosity curves at individual temperatures as well as for the master viscosity curves in Figure 2. Visually there is a good agreement between the experiments and the model. Based on this, the Carreau–Arrhenius model is capable of predicting the polymer viscosity with respect to shear rate and temperature.

### 3.2. Viscosity Curves of Drug/Polymer Mixtures

**Figure 3:**
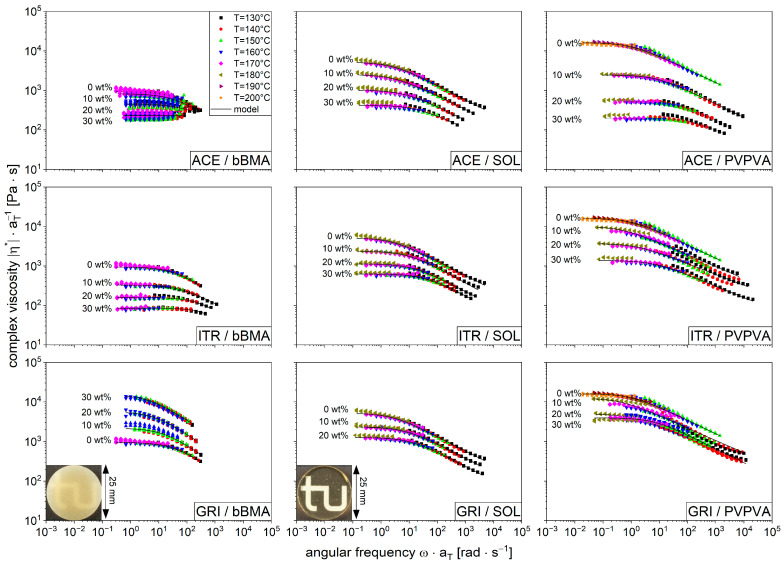
Master curves of the drug/polymer systems containing the drugs ACE (**top**), ITR (**middle**), and GRI (**bottom**) for the concentrations of 0 wt% to 30 wt% and the polymers bBMA (**left**), SOL (**middle**), and PVPVA (**right**) shifted to the reference temperature, T_ref_ = 160 °C. Enclosed visualization of polymer films 20 wt% GRI/bBMA and 20 wt% GRI/SOL.

The objective of this study was to characterize and model the influence of drugs on the viscosity of the polymer melts. For that purpose, three drug substances, acetaminophen (ACE), itraconazole (ITR), and griseofulvin (GRI), were combined with the aforementioned polymers (bBMA, SOL, and PVPVA) in various drug loads between 0 and 30 wt%. The complex viscosity was measured as a function of angular frequency and temperature (Figure 3). For visualization purposes, the viscosity curves of different melt temperatures were collapsed to the reference temperature (160 °C) using the time-temperature superposition concept as mentioned before. So, all flow curves of one composition are merged into one, which means all curves from Figure 1 are represented by the 0% functions in Figure 3. The resulting viscosity curves of the various drug loads are significantly different (α = 0.05, at zero shear rate viscosity), which was subsequently elaborated.

All formulations exhibit shear-thinning behavior; this is explained by the structural alignment of the polymers, as discussed before. Generally, the viscosities decrease with increasing drug content, causing a plasticizing effect on the drug substance for the specific polymer. The extent is highly specific due to a large difference in the pure drug/polymer properties, like glass transition temperature. A large difference leads to a strong impact on viscosity [45]. However, GRI had an anti-plasticizing effect on bBMA for all drug loads, which is why an increase in the viscosity with increased drug content was noticed. These sample specimens remained opaque during sample preparation, while the other samples turned transparent. Similar observations were obtained for 30 wt% GRI/SOL, and that is why these data are not shown. Furthermore, it could be demonstrated that each formulation for each specific drug content could be described by the aforementioned models (Equation (4), Figure 3, black lines). However, this approach is limited since each formulation has its own model parameters (Table A1, Table A2 and Table A3).

### 3.3. Modeling Function for Viscosity with Drug Shift Factor

Even if the influence of drug type and drug content on the melt viscosity could be described by the Carreau–Arrhenius model, different model parameters would be required for each drug/polymer ratio. This was seen as a shortcoming that constrains the applicability of this approach and was subsequently addressed. The new approach is based on the modified liquid-liquid mixing rule for polymers, which is applied to drug/polymer mixtures (Equation (5)) [30,46]. The rule gives the viscosity of a mixture $\left(\eta_{mix}\right)$ using the weight fractions $\left(w_{i}\right)$ and the viscosity of the pure drug $\left(\eta_{drug}\right)$ as well as the viscosity of the pure polymer $\left(\eta_{polymer}\right).$

(5) $$ln\left(\eta_{mix}\right)=\sum_{i}w_{i}\cdot ln\left(\eta_{i}\right)=w_{drug}\cdot ln\left(\eta_{drug}\right)+w_{polymer}\cdot ln\left(\eta_{polymer}\right)$$

Subsequently, a drug shift factor $\left(a_{drug}\left(w_{drug},w_{ref}\right)\right)$ was proposed, which considers the zero shear rate viscosity drug/polymer mixture at a given drug content $\left(\eta_{0}\left(w_{drug}\right)\right)$ and the zero shear rate viscosity of the pure polymer $\left(\eta_{0}\left(w_{ref}\right)\right)$ (Equation (6)).

(6) $$a_{drug}\left(w_{drug},w_{ref}\right)=\frac{\eta_{0}\left(w_{drug}\right)}{\eta_{0}\left(w_{ref}\right)}$$

The physical meaning of this drug shift factor is similar to the temperature shift factor, as discussed before. Generally, the content of a plasticizing drug exhibits similar behavior to a temperature increase, where high temperatures, as well as high percentages of a plasticizing drug, lower the viscosities for the shear rates. The drug shift factor is considered similarly to the temperature shift factor in Carreau’s approach (Equation (3)).

Through the combination of the drug shift factor (Equation (6)) with the liquid-liquid mixing rule (Equation (5)), Equation (7) is obtained, which still contains an expression with the pure drug viscosity $\left(\eta_{drug}\right)$ that is not readily accessible due to thermal degradation.

(7) $$a_{drug}\left(w_{drug},w_{ref}\right)=\exp\left(w_{drug}\cdot ln\left(\frac{\eta_{drug}}{\eta_{polymer}}\right)\right)=\exp\left(w_{drug}\cdot s_{drug}\right)$$

In order to determine this value, the drug shift factor is plotted on a logarithmic scale as a function of the drug content, and the slope $\left(s_{plast}\right)$ (Table A4) is given by $ln\left(\frac{\eta_{drug}}{\eta_{polymer}}\right)$. This viscosity relation parameter $\left(s_{plast}\right)$ finally expresses the effect on viscosity due to drug/polymer mixing. In the combined function, the drug shift factor was added in the established Carreau–Arrhenius approach (Equation (4)), and a global model in Equation (9) describes the viscosity as a function of shear rate, temperature, and drug content.

(8) $$\eta_{Modell}\left(\dot{\gamma },T,w_{drug}\right)=\frac{\eta_{0}\cdot a_{T}\left(T,T_{ref}\right)\cdot a_{drug}\left(w_{drug},w_{ref}\right)}{{\left(1+\frac{\dot{\gamma }}{{\dot{\gamma }}_{c}}\cdot a_{T}\left(T,T_{ref}\right)\cdot a_{drug}\left(w_{drug},w_{ref}\right)\right)}^{c}}$$

**Figure 4:**
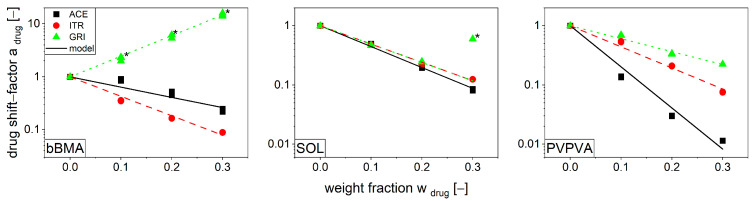
Drug shift factor for bBMA (**left**), SOL (**middle**), and PVPVA (**right**) including the fits for ACE (black-solid), ITR (red-dashed), and GRI (green-pointed) for the reference temperature, T_ref_ = 160 °C (*n* = 3). The data marked with * showed opaque drug/polymer films.

To illustrate the influence of the drug content on viscosity, the drug shift factor (Equation (6)), which is based on the previously shown relation of the zero shear rate viscosities (Table A1, Table A2 and Table A3), is plotted on a logarithmic scale against the drug content (Figure 4). For this purpose, the pure polymer without any drug served as a reference.

In general, a drug shift factor below 1 indicates a lower viscosity, while a drug shift factor above 1 indicates a higher viscosity when compared to the pure polymer. Thus, most formulations exhibit a plasticizing effect with an increase in drug content for various drugs, as previously discussed. Similar observations of the plasticizing effects of viscosity for various drugs were made by Bochmann [47]. The same experimental data indicate a nonlinear relationship between change in viscosity and drug weight fraction, as can be seen for ACE/bBMA and ACE/PVPVA. This can be attributed to a specific molecular repulsion between ACE and bBMA as well as an attraction between ACE and PVPVA [40]. This phenomenon was minor and could not be quantified with the experimental data. The substance system GRI/bBMA had a drug shift factor above 1 for all drug contents and exhibits a filler effect, as demonstrated by the fact that the sample specimens remained opaque throughout the experiment. With this filler effect, the drug/polymer mixture behaves as a suspension. This behavior can also be determined using the drug shift factor, with the difference that in a polymer suspension, the change in viscosity depends only on the volume fraction of the undissolved substance rather than on the viscosity of a specific drug.

Thus far, the drug shift factor is utilized to capture the rheological behavior if the drug is either dissolved or suspended in the polymer. However, in many practical applications, such as extrusion, mixing, or melt coating, there is an intermediate state that changes with respect to the drug polymer ratio. This behavior can be seen for GRI/SOL in Figure 4. For low weight fractions (0.1 and 0.2), a plasticizing effect is seen, resulting in a drug shift factor of less than 1. However, at a weight fraction of 0.3, the specimen is opaque, and the shift factor increases drastically with respect to 0.2. The rheological behavior of this system (30 wt% GRI/SOL) is dominated by a plasticizing and a filler effect at the same time, which was analyzed systematically.

**Figure 5:**
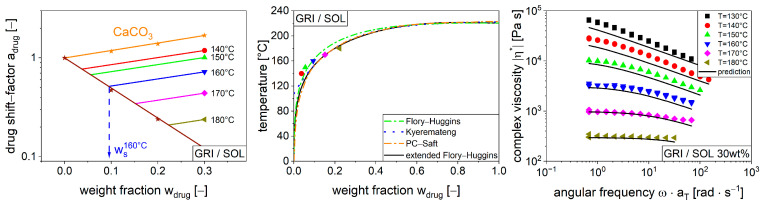
Drug shift factor for CaCO_3_/SOL as a filler system (orange stars) and GRI/SOL as a combined filler/plasticizing system (brown stars) with respect to drug content (**left**). Phase diagram for GRI/SOL with typical literature models (**center**), [40]. Complex viscosity as a function of angular frequency with the prediction from the phase diagrams for the system 30 wt% GRI/SOL (**right**).

Looking closer at the behavior of the 30 wt% GRI/SOL system, an effect of the temperature on the drug shift factors was seen. This phenomenon was unexpected since the temperature influence is already captured in the temperature shift factor. Drug shift factors were fitted for each temperature separately using Equation (6) (Figure 5, left, data points at w = 0.3). Moreover, the plasticizing effect of GRI on SOL was modelled using 0, 0.1, and 0.2 weight fractions as a linear function ($s_{plast}$, Figure 5, left, brown line). The filler effect was also quantified using the drug shift factor as a linear function of weight fraction $\left(s_{filler}\right)$. Therefore, calcium carbonate (CaCO_3_) was utilized since it is practically insoluble in SOL and the filler effect is rather drug independent, as mentioned previously (Figure 5, left, orange line). Since the influence of the temperature on polymer viscosity is captured in the temperature shift factor $\left(a_{T}\right)$ using time-temperature superposition, Figure 5 (left) refers to the reference temperature of 160 °C. The remaining temperature influence exists only for the suspended specimens and is attributed to differences in solubility. In fact, different temperatures yield different solubilities, resulting in different solid factions in the melt and different viscosities. Moving the suspension line (Figure 5, left, orange line) in parallel with the temperature-specific shift factors, generates an intersection with the plasticizing line (Figure 5, left, brown line). The weight fraction at the intersection is the solubility for a specific temperature (Figure 5, left, visualized for 160 °C).

The resulting temperature-dependent solubilities were compared to literature data [40,48] using a phase diagram (Figure 5, center). The common solubility models, such as Flory–Huggins [49], Kyeremateng [50], PC-SAFT [48], and the extended Flory–Huggins [40], have a certain variability in this region. In this respect, the experimental data fit quite nicely to the existing phase diagrams. However, there is systematic deviation from the solubility line at low weight fractions and temperatures. It is likely that the solubility is not in equilibrium in the rheology measurements since rather long equilibration times are expected in these highly viscous systems [51]. Nevertheless, it seems to be plausible to attribute the temperature influence on the drug shift factor to variations in solubility.

Based on this, it is possible to predict the temperature-specific drug shift factors from the phase diagram. Therefore, the extended Flory–Huggins–Solubility–line was utilized in accordance with the literature [40]. Considering a given temperature, it is possible to identify the solubility $\left(w_{s}\left(T\right)\right)$. This defines the ratio of the dissolved and suspended drug in the polymer melt and can be expressed as a drug shift factor $\left(a_{drug}\left(w_{drug},w_{s}(T)\right)\right)$, where $\left(w_{drug}\right)$ is the overall drug content of 0.3 in the case of the system 30 wt% GRI/SOL (Equation (9)).

(9) $$a_{drug}\left(w_{drug},w_{s}(T)\right)=\exp\left(w_{s}\left(T\right)\cdot s_{plast}+\left(w_{drug}-w_{s}\left(T\right)\right)\cdot s_{filler}\right)$$

When this is coupled to the other model parameters $\left(\eta_{0},{\dot{\gamma }}_{c},c,a_{T}\right)$ using Equation (8), the viscosity of a drug/polymer melt with a solid drug fraction can be predicted (Figure 5, right). Generally, this model can predict the experimental data, but systematic deviations were observed. These are likely due to the non-equilibrium measuring conditions in the rheological measurements, especially at lower temperatures.

The drug shift factor is an appropriate tool to incorporate drug content in the viscosity models of polymer melts. It is suitable to capture plasticizing and filler effects as well as intermediate states. It is straightforward to apply in common rheological models through a single factor.

## 4. Conclusions

The melt viscosity of nine common, binary, drug/polymer mixtures was characterized via oscillatory rheometry measurements utilizing vacuum compression molding. The repeatability was high, and results agreed with literature data.

Additionally, a model was developed to describe melt viscosity with respect to shear rate, temperature, drug content, and phase. Therefore, at least four rheological measurements are required to parametrize the model. The origin of this model is the Carreau approach, where the three parameters $\left(\eta_{0},{\dot{\gamma }}_{c},c\right)$ required to describe the shear-thinning behavior of the polymer are obtained by a single measurement. The second measurement is necessary to capture the temperature dependency of the polymer viscosity based on the Arrhenius activation energy $\left(E_{A}\right)$. There, the temperature must be altered with respect to the first measurement in order to elucidate the effect. Frequently the drugs have a plasticizing effect on the polymers, resulting in a decrease of viscosity with an increase in drug content. For low weight fractions, this effect was assumed to be linear and captured with a single parameter $\left(s_{plast}\right)$. This parameter can be determined by a further rheological measurement using a drug concentration that can be dissolved at a specific temperature. If the drug does not dissolve in the polymer, the viscosity increases, where the increase is independent from the drug substance itself. This behavior is also assumed to be linear with respect to the weight fraction $\left(s_{filler}\right)$. This parameter is assessed by a fourth rheological measurement utilizing an insoluble compound. Finally, the phase diagram of the drug-polymer mixture is required to quantify the dissolved and suspended drug fraction in the polymer melt.

The viscosity of the nine drug-polymer mixtures could be modeled with respect to shear rate, temperature, drug content, and phase using six model parameters that can be determined by just four rheological measurements per drug/polymer mixture. This model should be able to capture the rheological behavior of drug/polymer mixtures in various processes like mixing, extrusion, fused deposition modeling, or melt coating.

## Data Availability

The raw data supporting the conclusions of this article are included in the supplementary materials.

## Supplementary Materials

The following supporting information can be downloaded at: https://www.mdpi.com/article/10.3390/pharmaceutics16030301/s1, raw data of rotational and oscillatory viscosity measurements was provided.

## List of Symbols and Abbreviations

**Latin Symbols**	**Definition**	**Unit**
$a_{T}$	temperature shift factor	−
$a_{drug}$	drug shift factor	−
c	flow index	−
$E_{A}$	Arrhenius activation energy	$J\cdot {mol}^{-1}$
R	ideal gas constant	$J\cdot {mol}^{-1}\cdot K$
$s_{plast}$	drug relation parameter of a plasticizing drug	−
$s_{filler}$	drug relation parameter of filler	−
T	temperature	K
$T_{g}$	glass transition temperature	K
$T_{ref}$	reference temperature	K
$w_{drug}$	weight fraction of the drug	$kg\cdot {kg}^{-1}$
$w_{ref}$	reference weight fraction of the drug	$kg\cdot {kg}^{-1}$
$w_{s}$	weight fraction of the dissolved drug	$kg\cdot {kg}^{-1}$
**Greek Symbols**	**Definition**	**Unit**
$\dot{\gamma }$	shear rate	$s^{-1}$
${\dot{\gamma }}_{c}$	critical shear rate	$s^{-1}$
$\eta_{0}$	zero shear rate viscosity	Pa·s
$\eta \left(\dot{\gamma }\right)$	dynamic viscosity	Pa·s
$\left|\eta^{*}\right|\left(\omega \right)$	complex viscosity	Pa·s
ω	angular frequency	$rad\cdot s^{-1}$
**Abbreviations**	**Definition**	
ACE	acetaminophen	
bBMA	basic butylated methacrylate copolymer	
CaCO_3_	calcium carbonate	
GRI	griseofulvin	
ITR	itraconazole	
PVPVA	polyvinylpyrrolidone/vinyl acetate (copovidone)	
SOL	Soluplus	
Note: Tradenames and trademarks are used without particular labeling.		

## Appendix A

**Table A1:** Viscosity parameters of the Carreau-Arrhenius model of the polymer bBMA and the drugs ACE, ITR and GRI at a reference temperature, T_ref_ = 160 °C, as $\bar{x}\pm s$.

Drug	Content [wt%]	$\eta_{0}[Pa\cdot s]$	${\dot{\gamma }}_{c}[s^{-1}]$	c[−]	$E_{A}[J\cdot {mol}^{-1}]$
-	0	1011 ± 34	26.8 ± 5.5	0.433 ± 0.018	122,498 ± 801
ACE	10	806 ± 54	26.7 ± 6.0	0.400 ± 0.028	79,357 ± 1356
ACE	20	451 ± 46	30.0 ± 9.8	0.162 ± 0.033	43,055 ± 1883
ACE	30	179 ± 22	77.4 ± 1.2	0.117 ± 0.028	44,991 ± 2784
ITR	10	343 ± 7	72.8 ± 13.4	0.437 ± 0.027	115,075 ± 480
ITR	20	158 ± 3	369.4 ± 98.8	0.581 ± 0.098	114,195 ± 1198
ITR	30	86 ± 1	253.56 ± 49.8	0.368 ± 0.044	1,113,365 ± 865
GRI	10	2172 ± 159	14.8 ± 1.8	0.495 ± 0.009	110,747 ± 6318
GRI	20	5607 ± 462	7.1 ± 1.0	0.484 ± 0.015	110,022 ± 5735
GRI	30	14,928 ± 1 386	4.5 ± 1.1	0.467 ± 0.020	132,317 ± 6475

**Table A2:** Viscosity parameters of the Carreau-Arrhenius model of the polymer SOL and the drugs ACE, ITR and GRI at a reference temperature, T_ref_ = 160 °C, as $\bar{x}\pm s$.

Drug	Content [wt%]	$\eta_{0}[Pa\cdot s]$	${\dot{\gamma }}_{c}[s^{-1}]$	c[−]	$E_{A}[J\cdot {mol}^{-1}]$
-	0	5146 ± 93	2.5 ± 0.1	0.363 ± 0.002	130,846 ± 344
ACE	10	2484 ± 55	3.1 ± 0.2	0.349 ± 0.001	123,170 ± 405
ACE	20	1003 ± 30	7.3 ± 0.7	0.351 ± 0.004	117,018 ± 420
ACE	30	424 ± 11	17.0 ± 2.7	0.322 ± 0.009	113,743 ± 595
ITR	10	2410 ± 16	7.7 ± 0.3	0.380 ± 0.004	128,483 ± 230
ITR	20	1123 ± 22	14.3 ± 1.2	0.368 ± 0.003	129,862 ± 585
ITR	30	631 ± 6	33.8 ± 1.1	0.371 ± 0.004	131,218 ± 604
GRI	10	2326 ± 39	6.8 ± 0.4	0.362 ± 0.004	131,394 ± 304
GRI	20	1237 ± 11	13.0 ± 0.4	0.373 ± 0.002	138,862 ± 520
GRI	30	2987 ± 40	15.4 ± 1.0	0.456 ± 0.005	181,353 ± 816
CaCO_3_	10	5497 ± 15	4.0 ± 0.1	0.389 ± 0.002	159,612 ± 164
CaCO_3_	20	6616 ± 12	3.9 ± 0.1	0.389 ± 0.002	162,443 ± 1 004
CaCO_3_	30	8191 ± 47	3.5 ± 0.1	0.391 ± 0.004	161,034 ± 525

**Table A3:** Viscosity parameters of the Carreau-Arrhenius model of the polymer PVPVA and the drugs ACE, ITR and GRI at a reference temperature, T_ref_ = 160 °C, as $\bar{x}\pm s$.

Drug	Content [wt%]	$\eta_{0}[Pa\cdot s]$	${\dot{\gamma }}_{c}[s^{-1}]$	c[−]	$E_{A}[J\cdot {mol}^{-1}]$
-	0	18,296 ± 486	1.0 ± 0.0	0.368 ± 0.003	184,119 ± 2442
ACE	10	2432 ± 44	8.4 ± 0.4	0.348 ± 0.003	161,786 ± 377
ACE	20	534 ± 10	37.2 ± 2.8	0.333 ± 0.006	146,410 ± 656
ACE	30	202 ± 1	89.4 ± 0.6	0.286 ± 0.001	145,155 ± 388
ITR	10	9596 ± 268	0.6 ± 0.0	0.307 ± 0.002	193,820 ± 1706
ITR	20	3697 ± 60	1.5 ± 0.1	0.275 ± 0.002	182,274 ± 458
ITR	30	1333 ± 12	6.6 ± 0.7	0.278 ± 0.003	178,054 ± 984
GRI	10	12,303 ± 96	0.4 ± 0.0	0.310 ± 0.003	217,872 ± 1567
GRI	20	5872 ± 78	0.3 ± 0.0	0.258 ± 0.002	212,188 ± 1318
GRI	30	3965 ± 25	6.2 ± 0.7	0.351 ± 0.002	211,522 ± 1088

**Table A4:** Linear correlation of the influence of viscosity for the additive/polymers by the viscosity relation parameter $s_{plast}$ and $s_{filler}$ as $\bar{x}\pm s$.

Additive/Polymer	bBMA	SOL	PVPVA
ACE	−4.47 ± 0.37	−8.11 ± 0.11	−15.98 ± 0.05
ITR	−8.49 ± 0.07	−7.11 ± 0.05	−8.19 ± 0.05
GRI	8.97 ± 0.34	−7.13 ± 0.09	−5.02 ± 0.02
CaCO_3_	-	1.51 ± 0.01	-
